# Zerumbone, a Natural Cyclic Sesquiterpene of *Zingiber zerumbet* Smith, Attenuates Nonalcoholic Fatty Liver Disease in Hamsters Fed on High-Fat Diet

**DOI:** 10.1155/2013/303061

**Published:** 2013-10-09

**Authors:** Thing-Fong Tzeng, Shorong-Shii Liou, Chia Ju Chang, I-Min Liu

**Affiliations:** Department of Pharmacy & Graduate Institute of Pharmaceutical Technology, Tajen University, Yanpu Township, Pingtung County 90701, Taiwan

## Abstract

We investigated the effects of zerumbone, a natural cyclic sesquiterpene, on hepatic lipid metabolism in Syrian golden hamsters fed on high-fat diet (HFD). After being fed HFD for 2 weeks, hamsters were dosed orally with zerumbone (75, 150, and 300 mg kg^−1^) once daily for 8 weeks. After treatment with zerumbone, the plasma levels of total cholesterol (TC) and triglycerides (TGs) and the contents of TC and TG in hepatic tissue as well as homeostasis model assessment of insulin resistance were lowered, especially in the zerumbone-treated group (300 mg kg^−1^). Moreover, the histological evaluation of liver specimens demonstrated that the steatosis and inflammation in liver of zerumbone-treated groups were improved. Zerumbone exhibited the ability to decrease hepatic mRNA levels of sterol regulatory element-binding protein-1c and its lipogenic target genes, such as fatty acid synthase, acetyl-CoA carboxylase 1, and stearoyl-CoA desaturase 1. The hepatic mRNA expression of peroxisome proliferator-activated receptor **α**, together with its target genes including carnitine palmitoyl transferase-1, acyl-CoA oxidase, and acyl-CoA oxidase 1, was also upregulated by zerumbone. In conclusion, zerumbone improves insulin sensitivity, decreases lipogenesis, and increases lipid oxidation in the liver of HFD-fed hamsters, implying a potential application in the treatment of nonalcoholic fatty liver disease.

## 1. Introduction

Nonalcoholic fatty liver disease (NAFLD) represents a wide spectrum of diseases, ranging from simple fatty liver (hepatic steatosis) through steatosis with inflammation and necrosis to cirrhosis [1]. NAFLD, which is strongly associated with obesity, insulin resistance, and type 2 diabetes, is now well recognized as being part of the metabolic syndrome [2]. The metabolic pathways leading to the development of hepatic steatosis are multiple, including enhanced nonesterified fatty acid release from adipose tissue (lipolysis), increased *de novo* fatty acids (lipogenesis) and decreased *β*-oxidation. To date, caloric restriction and aerobic exercise are the effective treatments of NAFLD, but they are difficult to achieve for most NAFLD patients. Statins are the first-line agents to treat hyperlipidaemia, but there is a risk for liver injury in patients with NAFLD [3]. The most promising pharmacological treatment of NAFLD is peroxisome proliferator-activated receptor *α* (PPAR) *α* agonist, such as lipanthyl, that decreases lipid accumulation in hepatocytes [4], while the adverse effects may occur in some patients [5]. Recently, sterol regulatory element-binding protein-1c (SREBP-1c) has been highly evaluated as a potential target for the treatment of NAFLD, based on its advances to control lipogenic gene expression and regulate fatty acid and triglyceride (TG) homeostasis [6, 7]. Thus, inhibition of hepatic SREBP-1c signaling pathway could improve dyslipidemia and NAFLD. Until now, no specific pharmacological treatment for liver steatosis has been defined [8]. Therefore, the development of additional therapies for controlling lipid levels is warranted to attenuate hepatic steatosis.

*Zingiber zerumbet* Smith is one kind of plant growing mainly in Southeast Asia, which has been demonstrated to possess antinociceptive, anti-inflammatory, antiulcer, antihyperglycemic, and antiplatelet activities [9]. As a major compound extract, zerumbone ((2*E*,6*E*,10*E*)-2,6,9,9-tetramethylcycloundeca-2,6,10-trien-1-one) is currently explored for its potential broad use on cancers and leukemia, as well as virus infection [10–12]. Several studies have shown that zerumbone also produced a variety of pharmacological effects, including antioxidants, anti-inflammatory, and antibacterial [13, 14]. Our previous studies have indicated that *Z. zerumbet* might reduce the TG level in plasma and hepatic tissues in high-fat-diet-(HFD-) induced rats [15]; whether the effects of *Z. zerumbet* is mediated by zerumbone remained not clear.

The effects of dietary cholesterol and fat on plasma lipid profiles are similar in hamsters and humans, and fatty liver and mild diabetes were developed in hamsters after fed HFD [16]. The hamsters fed with HFD may thus be a good animal model for research on the treatment of diet-induced metabolic syndrome complicated by NAFLD [17]. Thus, the aim of this work is to assess the effects of zerumbone on preventing hepatic lipid accumulation in HFD-induced NAFLD hamsters. To gain further insights into the molecule mechanism by which zerumbone alters gene expression of hepatic lipids metabolism, the mRNA expression of SREBP-1c and its response genes were also determined. Besides the genes involved in fatty acid *de novo* synthesis, the genes involved in fatty acid *β*-oxidation, like PPAR*α* and its target genes, were also measured in this study. 

## 2. Materials

### 2.1. Animal Models and Treatment Protocols

Male Golden Syrian hamsters, 8-week old and weighing 90 ± 10 g, were obtained from the National Laboratory Animal Center (Taipei, Taiwan). They were maintained in a temperature-controlled room (25 ± 1°C) on a 12 h : 12 h light-dark cycle (lights on at 06:00 h) in in our animal center. Food and water were provided *ad libitum*. A regular chow diet (RCD; 10% kcal fat, no. D12450B, Research Diets, New Brunswick, NJ, USA) was used as the maintenance and control diet. A purified HFD with 45% kcal fat obtained primarily from lard (no. D12451, Research Diets) was used to induce a rapid increase in body weight and obesity [18]. All animal procedures were performed according to the Guide for the Care and Use of Laboratory Animals of the National Institutes of Health, as well as the guidelines of the Animal Welfare Act. These studies were conducted with the approval of the Institutional Animal Care and Use Committee (IACUC) at Tajen University (approval number: IACUC 100-29; approval date: December 22, 2011). 

After being fed a HFD for two weeks, hamsters were dosed by oral gavage once per day for eight weeks with zerumbone (≥98%; Sigma-Aldrich, St. Louis, MO, USA) doses of 75, 150, and 300 mg/kg in a volume of 1.5 mL kg^−1^ distilled water. Another group of HFD-fed hamsters was treated orally for eight weeks with 100 mg kg^−1^ per day lipanthyl (Laboratories Fournier SA, France). The dose of fenofibrate was based on study that has documented that long-term fenofibrate treatment could ameliorate hepatic insulin resistance and steatosis in high fructose-fed mice [4]. Another group of HFD- and RCD-fed rats were treated similarly, but the same volume of vehicle (distilled water) was used to prepare the tested compound solutions during the same treatment period. The water consumption, food intake, and body weight were measured once daily at the same time (09 : 00) on each day throughout the experiment. Food cups containing fresh food each time were weighed at the beginning and end of each 24 h period. Food intake was calculated by determining the difference in food cup weights, adjusting for any spillage that occurred. Water intake was calculated by measuring the difference in water bottle weights at the beginning and end of the daily change of water.

Eight weeks after treatment with zerumbone (total diet-fed period was 10 weeks), animals were weighed and anesthetized with ketamine after fasting for 12 hours. Blood samples were taken from the inferior vena cava in order to determine the plasma biomarkers. After collecting the blood, the liver was removed, rinsed with a physiological saline solution, and immediately stored at −70°C. The coefficient of hepatic weight was also calculated as liver weight (g) divided by body weight (100 g).

### 2.2. Determination of Metabolic Parameters and Insulin Sensitivity

Blood samples were centrifuged at 2,000 ×g for 10 minutes at 4°C. The plasma was then removed and placed into aliquots for the respective analytical determinations. Kits for determining plasma glucose (Cat. no. 10009582) concentration were purchased from Cayman Chemical Company (Ann Arbor, MI, USA). Commercial enzyme-linked immunosorbent assay (ELISA) kits were used to quantify plasma insulin concentration (LINCO Research, Inc. St. Charles, MO, USA; Cat. no. EZRMI-13K). The diagnostic kits for determinations for plasma levels of total cholesterol (TC; Cat. no. 10007640) and TG (Cat. no. 10010303) were purchased from Cayman Chemical Company (Michigan, USA). The diagnostic kit for determinations for plasma levels of high density lipoprotein cholesterol (HDL-C) was purchased from Bio-Quant Diagnostics (CA, USA; Cat. no. BQ 019CR), low density lipoprotein cholesterol (LDL-C) was calculated by using Friedewald's equation [19]. Plasma free fatty acid (FFA) levels were determined using an FFA quantification kit obtained from Abcam plc (MA, USA; Cat. no. ab65341). All experimental assays were carried out according to the manufacturer's instruction. All samples were analyzed in triplicate. 

Whole-body insulin sensitivity was estimated using the homeostasis model assessment of insulin resistance (HOMA-IR) by using the formula: (fasting serum glucose (mmol) times fasting serum insulin (mU mL^−1^))/22.5 [20]. Because HOMA is negatively correlated with insulin sensitivity, low HOMA-IR values indicate high insulin sensitivity, whereas high HOMA-IR values indicate insulin resistance.

### 2.3. Measurement of Hepatic Lipids

After removal from animals, sections of fresh liver samples were collected for determining the lipid content. Liver (1.25 g) was homogenized with chloroform/methanol (1 : 2, 3.75 mL), and then chloroform (1.25 mL) and distilled water (1.25 mL) were added to the homogenate and mixed well together. After centrifugation (1,500 ×g for 10 min), the lower clear organic phase solution was transferred into a new glass tube and then lyophilized. The lyophilized powder was dissolved in chloroform/methanol (1 : 2) and stored at −20°C for less than 3 days [21]. Hepatic TC and TG levels in lipid extracts were analyzed with the diagnostic kits that were used in the plasma analysis.

### 2.4. Histological Staining and Oil Red O Staining

For histopathological analysis, the sections of liver were fixed in 10% formalin, dehydrated, embedded in paraffin, and stained with hematoxylin and eosin (H&E). For oil red O (ORO) staining, a stock solution of oil red O (0.5 g per 100 mL) in isopropanol was prepared, stored, and protected from light. Liver tissue was embedded in optimal cutting temperature gel. Air dried tissue sections of 5 *μ*m were dipped in formalin, washed with ORO without counterstaining with hematoxylin. All slides were scanned at an absolute magnification of 200x using Image Pro Plus 7.0 software (Media Cybernetics) under a light microscopy (Olympus, BX51 microscope, Tokyo, Japan).

### 2.5. Analysis of mRNA Expression of Hepatic Genes

For analysis of gene expression, total RNA was extracted from 100 mg frozen liver samples using Trizol reagent (Invitrogen, USA). RNA was quantified by A260 and its integrity verified by agarose gel electrophoresis using ethidium bromide for visualization. For the RT reaction, 1 *μ*g of total RNA per sample and 8.5 *μ*g *μ*L^−1^ random hexamer primers were heated at 65°C for 5 min and then quenched on ice. This mixture was combined with 500 *μ*mol L^−1^ each of dATP, dTTP, dCTP, and dGTP, 10 mmol L^−1^ DTT, 20 mmol L^−1^ Tris-HCl (pH 8.4), 50 mmol L^−1^ KCl, 5 mmol L^−1^ MgCl_2_, 40 units of RNaseOUT Recombinant Ribonuclease Inhibitor (Invitrogen, Boston, MA, USA), and 100 units SuperScript III reverse transcriptase (Invitrogen). Samples were subjected to DNase (Promega, Madison, WI, USA) treatment at 37°C for 20 min in a GeneAmp 9700 Thermal Cycler (Applied Biosystems, California, USA) and then held at 4°C. After aliquots were taken for immediate use in the PCR, the remainder of the cDNA was stored at −20°C. The mRNA expressions were measured by quantitative real-time RT-PCR in a fluorescent temperature Lightcycler 480 (Roche Diagnostics, Mannheim, Germany). The SREBP-1c primers sequences were as follows: forward, 5′-CGCTACCGTTCCTCTATCAA-3′; reverse, 5′-TTCGCAGGGTCAGGTTCTC-3′. The acetyl-CoA carboxylase 1 (ACC1) primers were as follows: forward, 5′-GGACAGAC1GATCGCAGAGAAAG-3′; reverse, 5′-TGGAGAGCCCCACACACA-3′. The fatty acid synthase (FAS) primers were as follows: forward, 5′-GGAACTGAACGGCATTACTCG-3′; reverse, 5′-CATGCCGTTATCAACTTGTCC-3′. The stearoyl-CoA desaturase (SCD)1 primers were as follows: forward, 5′-CCTTAACCCTGAGATCCCGTAGA-3′; reverse, 5′-AGCCCATAAAAGATTTC1GCAAA-3′. The PPAR*α* primers sequences were as follows: forward, 5′-GAAGCAGATGACCTGGAAAGT-3′; reverse, 5′-AGCCTGGACAGCTCCCTAA-3′. The carnitine palmitoyl transferase (CPT)-1 primers sequences were as follows: forward, 5′-GCTTCCCCTTACTGGTTCC-3′; reverse, 5′-AACTGGCAGGCAATGAGACT-3′. The acyl-CoA oxidase (ACO) primers sequences were as follows: forward, 5′-ACTATATTTGGCCAATTTTGTG-3′; reverse, 5′-TGTGGCAGTGGTTTCCAAGCC-3′. The acyl-CoA oxidase 1 (ACOX1) primers sequences were as follows: forward, 5′-GTTGATCACGCACATCTTGGA-3′; reverse, 5′-TCGTTCAGAATCAAGTTCTCAATTTC-3′. The tumor necrosis factor (TNF)-*α* primers sequences were as follows: forward, 5′-ACACCATGAGCACGGAAAGC-3′; reverse, 5′-CCGCCACGAGCAGGAA-3′. The interleukin (IL)-1*β* primers sequences were as follows: forward, 5′-AATGGACAGAACATAAGCCAACA-3′; reverse, 5′-CCCAAGGCCACAGGGAT-3′. The *β*-actin primers sequences were as follows: forward, 5′-TCACCCACACTGTGCCCATCTA-3′; reverse, 5′-TTGCTGATCCACATCTGCTGG-3′. Primers were designed with Primer Express Software version 2.0 System (Applied Biosystems, Foster City, CA, USA). T PCR reaction was performed following the cycling protocol of 95°C for 5 min, followed by 45 PCR cycles with 95°C for 5 s, 58°C for 15 s and 72°C for 20 s. Dissociation curves were run after amplification to identify the specific PCR products. The mRNA expression levels were normalized by the *β*-actin mRNA levels and calculated according to the delta-delta Ct method [22]. 

### 2.6. Statistical Analysis

Data are expressed as the mean ± standard deviation (SD). Statistical analysis was performed with one-way analysis of variance (ANOVA). Dunnett range post hoc comparisons were used to determine the source of significant differences, where appropriate. A *P* value <.05 was considered statistically significant.

## 3. Results

### 3.1. Effects of Treatments on the Body Weight, the Relative Liver Weights, and Feeding Behaviors of Hamsters

At the end of 8-week treatment, the body weight and relative liver weights in HFD-fed hamsters were significantly increased over those of RCD-fed group (Table 1). Zerumbone significantly suppressed body weight gain at high doses (300 mg kg^−1^ per day). The coefficient of hepatic weight *i* zerumbone-treated HFD-fed hamsters (300 mg kg^−1^ per day) was significantly lower than that of the vehicle-treated group. Similar results were seen in HFD-fed hamsters treated with lipanthyl (100 mg kg^−1^ per day, Table 1). No significant differences in daily food and water intake were observed among the groups over the experimental period (Table 1).

### 3.2. Effects of Treatments on Plasma Lipids Levels and Insulin Sensitivity of Hamsters

The HFD caused elevated concentrations of plasma TC, TG, and LDL-C. The moderate (150 mg kg^−1^ per day) and high doses (300 mg kg^−1^ per day) of zerumbone significantly reduced plasma total TC levels (14.9% and 17.1% reduction, resp.) compared with vehicle-treated, HFD-fed hamsters (Table 1). All doses of zerumbone decreased plasma TG levels in HFD-fed hamsters (Table 1). The low, moderate, and high doses of zerumbone significantly reduced plasma LDL-C levels (6.2%, 16.7%, and 22.5% reductions, resp.; Table 1). Plasma TC, TG, and LDL-C concentrations were reduced significantly by 20.6%, 49.6%, and 28.2%, respectively, in lipanthyl-treated HFD-fed hamsters compared with vehicle-treated HFD-fed hamsters (Table 1).

The plasma concentration of HDL-C in HFD-fed hamsters was reduced to 27.3% of the level in the RCD-fed group (Table 1). After 8 weeks of treatment with zerumbone (300 mg kg^−1^ per day) or lipanthyl, the plasma HDL-C concentration in HFD-fed hamsters was elevated to nearly that of the RCD-fed group (Table 1).

Plasma FFAs were significantly higher in vehicle-treated HFD-fed hamsters compared to RCD-fed rats (Table 1). The plasma FFA level was reduced by 35.4% in HFD-fed hamsters treated with zerumbone (100 mg kg^−1^ per day) compared with their vehicle-treated counterparts (Table 1). Lipanthyl treatment reduced FFA concentrations in HFD-fed hamsters by 41.5% relative to the level in vehicle-treated HFD-fed hamsters (*P* < .05; Table 1).

HFD-fed hamster was insulin resistant reflected by hyperinsulinemia as well as significantly increased value of HOME-IR (Table 1). Treatment HFD-fed hamster with zerumbone (300 mg kg^−1^ per day) produced a similar effect to lipanthyl on insulin resistance, evidenced by reduction of fasting serum insulin levels and improved HOME-IR (Table 1).

### 3.3. Effects of Treatments on Hepatic Steatosis

The hepatic TC level was significantly higher in HFD-fed hamsters than in hamsters from the RCD-fed group, which was reduced by 23.8% in HFD-fed hamsters treated with zerumbone (300 mg kg^−1^ per day; Table 1). Similarly, zerumbone treatment (300 mg kg^−1^ per day) also produced a significant reduction in hepatic TG concentration to 64.8% of that in vehicle-treated, HFD-fed hamsters (Table 1). Hepatic TC and TG levels were significantly reduced (by 35.2% and 41.7%, resp.) in lipanthyl-treated HFD-fed hamsters compared with vehicle-treated counterparts (Table 1).

**Figure 1:**
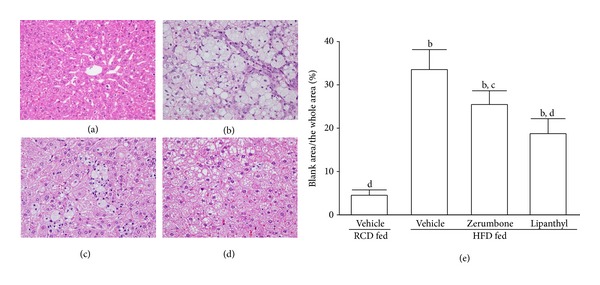
Representative images of H&E stain in livers from RCD- or HFD-fed hamsters receiving 8-week treatment. Photomicrographs are of tissues isolated from vehicle-treated RCD-fed hamsters (a), vehicle-treated HFD-fed hamsters (b), zerumbone-treated HFD-fed hamsters (300 mg kg^−1^ per day) (c), or lipanthyl-treated HFD-fed hamsters (100 mg kg^−1^ per day) (d). Photomicrographs were taken at a magnification of 200x. The quantification of the hepatic lipid droplets accumulation was presented as the percentage of the blank area (lipid droplets) relative to the whole area of the photomicrograph (e). Values (mean ± SD) were obtained from each group of 5 animals in each group. ^b^ *P* < .01 compared to the values of vehicle-treated RCD-fed hamsters. ^c^ *P* < .05 and ^d^ *P* < .01 compared to the values of vehicle-treated HFD-fed hamsters in each group, respectively.

The photomicrographs of the H&E stain showed that HFD feeding increased hepatic fat deposits, evidenced by the majority of the hepatocytes of HFD-fed hamsters that were distended by fat in comparison to the RCD-fed group (Figures 1(a) and 1(b)). The images of H&E stain also displayed macrovesicular steatosis in the hepatocytes of HFD-fed hamsters, as many single large droplets had displaced the nucleus and ballooning degeneration causing conspicuous swelling of the cell and cytoplasmic vacuolation (Figure 1(b)). The treatment of HFD-fed hamsters with zerumbone or lipanthyl reduced fat liver depots and less macrovesicular steatosis as revealed in vehicle-treated counterparts (Figures 1(c) and 1(d)). 

**Figure 2:**
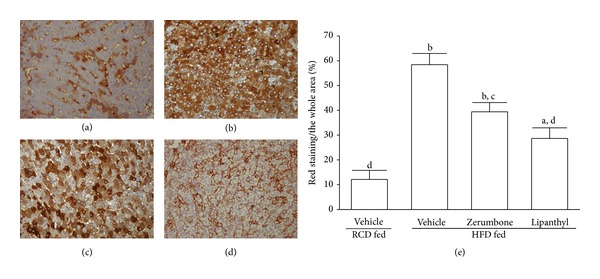
Representative images of ORO staining in livers from RCD- or HFD-fed hamsters receiving 8-week treatment. Photomicrographs are of tissues isolated from vehicle-treated RCD-fed hamsters (a), vehicle-treated HFD-fed hamsters (b), zerumbone-treated HFD-fed hamsters (300 mg kg^−1^ per day) (c), or lipanthyl-treated HFD-fed hamsters (100 mg kg^−1^ per day) (d). Photomicrographs were taken at a magnification of 200x. The quantification of the hepatic lipid droplets accumulation was presented as the percentage of the red staining area (lipid droplets) relative to the whole area of the photomicrograph (e). Values (mean ± SD) were obtained from each group of 5 animals in each group. ^a^ *P* < .05 and ^b^ *P* < .01 compared to the values of vehicle-treated RCD-fed hamsters in each group, respectively. ^c^ *P* < .05 and ^d^ *P* < .01 compared to the values of vehicle-treated HFD-fed hamsters in each group, respectively.

ORO staining on frozen liver sections exhibited many lipid droplets in liver sections of HFD-fed hamsters (Figure 2(b)), whereas few lipid droplets were seen in the liver sections from the RCD-fed (Figure 2(a)) and lipanthyl-treated HFD-fed hamsters (Figure 2(d)). Similarly, there was a decrease in the lipid content of liver tissue in zerumbone-treated HFD-fed hamsters (300 mg kg^−1^ per day) compared with vehicle-treated counterparts (Figure 2(c)). Analysis of blindly scored ORO-stained sections showed a statistically significant increase in the lipid content of liver tissue of HFD-fed hamsters (Figure 2(e)). Levels of lipids were higher in the liver of HFD-fed hamsters (58.4 ± 4.5%) compared with those in RCD-fed group (12.1 ± 3.6%). Hepatic lipids values were reduced significantly by 32.6%, and 50.7%, respectively, in zerumbone-treated HFD-fed hamsters (300 mg kg^−1^ per day) or lipanthyl-treated HFD-fed hamsters compared with vehicle-treated counterparts.

### 3.4. Effects of Treatments on Hepatic mRNA Expression of SREBP-1c and Its Lipogenic Target Genes in Hamsters

**Figure 3:**
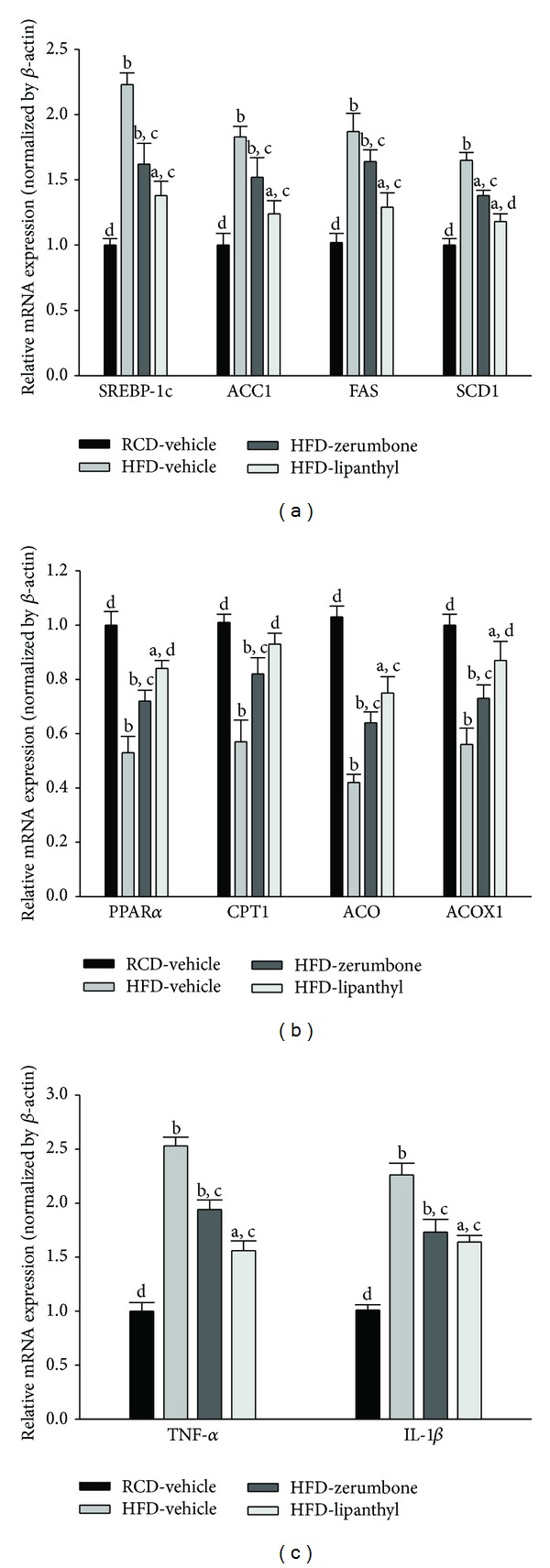
The hepatic mRNA levels of SREBP-1c and its lipogenic target genes (a), PPAR*α* and its target genes responsible for fatty acid *β*-oxidation (b), and inflammatory cytokines (c) in RCD- or HFD-fed hamsters receiving 8-week treatment with zerumbone (300 mg kg^−1^ per day, HFD-zerumbone) or lipanthyl (100 mg kg^−1^ per day, HFD-lipanthyl). The mRNA expressions of the lipogenic genes were measured by RT-PCR and normalized to an internal control (*β*-actin). Animals not receiving any treatment were given the same volume of vehicle used to dissolve zerumbone. Similar results were obtained with an additional 4 replications. Data were expressed as the mean with SD (*n* = 5 per group) in each column. ^a^ *P* < .05 and ^b^ *P* < .01 compared to the values of vehicle-treated RCD-fed hamsters (RCD-vehicle). ^c^ *P* < .05 and ^d^ *P* < .01 compared to the values of vehicle-treated HFD-fed hamsters (HFD-vehicle).

HFD feeding markedly increased the hepatic mRNA levels of SREBP-1c in hamsters to 2.2-fold relative to those in RCD-fed group (Figure 3(a)). Hepatic mRNA levels of SREBP-1c were significantly reduced (by 38.1%) in lipanthyl-treated HFD-fed hamsters compared with the vehicle-treated counterparts (Figure 3(a)). Zerumbone (300 mg kg^−1^ per day) suppressed the HFD-induced stimulation in hepatic mRNA levels of SREBP-1c to 72.6% relative to those in their vehicle-treated counterparts (Figure 3(a)).

HFD caused a 1.9-fold induction of hepatic ACC1 mRNA, a 2.0-fold induction of hepatic FAS mRNA, and a 1.7-fold induction of hepatic SCD1 mRNA relative to those in RCD-fed group. The HFD-induced mRNA levels of ACC1, FAS, and SCD1 in liver were significantly reversed after lipanthyl treatment (by 35.8, 37.9, and 28.5% decrease, resp.) compared to those of the vehicle-treated counterparts (Figure 3(a)). The hepatic mRNA levels of ACC1, FAS, and SCD1 were downregulated by zerumbone (300 mg kg^−1^ per day) treatment, with a decrease of 21.2, 21.1, and 16.4%, respectively, when compared with those observed in the vehicle-treated counterparts (Figure 3(a)).

### 3.5. Effects of Treatments on Hepatic mRNA Expression of PPAR*α* and Its Target Genes Responsible for Fatty Acid *β*-Oxidation in Hamsters

The mRNA levels of PPAR*α* in livers of HFD-fed hamsters were lower to 53.1% of those from RCD-fed group (Figure 3(b)). Administration HFD-fed hamsters with lipanthyl or zerumbone (300 mg kg^−1^ per day) for 8 weeks significantly upregulated the hepatic PPAR*α* mRNA levels to 1.4- and 1.6-fold relative to those in vehicle-treated counterparts, respectively (Figure 3(b)). 

Compared with RCD-fed group, the hepatic mRNA levels of CPT-1, ACO, and ACOX1 in HFD-fed hamsters decreased obviously, which were up-regulated by lipanthyl treatment (155.3, 178.6, and 155.3% increases, resp.) (Figure 3(b)). The hepatic mRNA levels of CPT-1, ACO, and ACOX1 in HFD-fed hamsters receiving zerumbone (300 mg kg^−1^ per day) treatment were increased to 143.8, 152.4, and 130.4% relative to the expression levels in vehicle-treated counterparts, respectively (Figure 3(b)). 

### 3.6. Effects of Treatments on Hepatic mRNA Expression of Inflammatory Cytokines in Hamsters

In HFD hamsters, TNF-*α* and IL-1*β* were significantly increased around 2.5- and 2.3-fold, respectively, as compared to that of RCD-fed group. Administration of HFD-fed hamsters with lipanthyl for 8 weeks significantly downregulated the hepatic TNF-*α* and IL-1*β* mRNA levels to 61.6 and 72.6% relative to those in vehicle-treated counterparts, respectively (Figure 3(c)). Zerumbone (300 mg kg^−1^ per day) treatment reversed HFD-induced increase of TNF-*α* and IL-1*β* mRNA expression to 76.6 and 76.5% of those observed in the vehicle-treated counterparts, respectively (Figure 3(c)). 

## 4. Discussion

The pathogenic mechanisms of NAFLD are still under investigation; however, fat accumulation, mainly TG filtration within hepatocytes, is considered the first step in the development of NAFLD [1]. The clinical and animal studies demonstrated that levels of hepatic TG are positively correlated to visceral obesity and insulin-resistance [23]. Under insulin resistant status, FFAs from lipolysis of visceral tissue are increased with decreased oxidative capacity. The elevated FFAs in the blood stream will directly circulate into the portal vein where the liver deposits FFAs as TG in the hepatocytes and contributes to liver fibrosis [8]. Therefore, aggressive treatment of hyperlipidaemia plays a critical role in the overall management of patients with NAFLD [8]. HFD-induced animal model of NAFLD has been widely used to identify the pathogenesis and evaluate new treatment for NAFLD [17]. In our model, HFD-fed hamsters developed hepatic steatosis, visceral obesity, and hyperlipidaemia and increased FFA and HOMR-IR values, which mimics almost all of the clinical aspects of human NAFLD [24]. We observed that the increased levels of  TG, TC, LDL-C, and FFA in the plasma were significantly suppressed, whereas the decreased plasma HDL-C levels were obviously elevated with zerumbone treatment in HFD-fed hamsters. We showed that zerumbone had effects on hypertriglyceridemia and attenuated the elevated plasma FFAs, indicating a potential application of zerumbone in treating fatty liver diseases.

TG is thought to be a surrogate marker of disrupted insulin signal. In other words, hepatic insulin resistance is associated with the accumulation of TG and fatty acid metabolites [25]. Zerumbone also presented similar effects to lipanthyl on hyperinsulinemia and improved HOME-IR, although its potency was much less than lipanthyl as the higher dose (300 mg kg^−1^ per day) of zerumbone than lipanthyl (100 mg kg^−1^ per day) was needed. The raised insulin sensitivity could also reflect less lipid accumulation in the liver indirectly.

Decrease of hepatic lipid accumulation is a rational target for NAFLD therapy since lipid loss should reduce many of the putative mediators of liver injury including insulin resistance, hepatic FFA supply and pro-inflammation [24]. Zerumbone treatment significantly decreased hepatic TG content. In addition, the relative liver weight in zerumbone treated hamsters were significantly lower than that of HFD-fed hamsters. Morphologically, the liver of HFD-fed hamsters showed abundant and large lipid droplets and obvious increase of liver derangement compared to that of RCD-fed hamsters. However, the liver of HFD-fed hamsters receiving zerumbone had fewer lipid droplets and more normal liver morphology, suggesting a beneficial effect of zerumbone on preventing lipid accumulation and reversal of disrupted structure of the liver. Zerumbone may also exert liver protective effect via inhibition of HFD-induced inflammation, since our results showed that zerumbone decreased gene expression of inflammatory cytokines resulting from lipid accumulation in liver [26].

To explore the possible mechanisms of zerumbone on decreasing hepatic lipids accumulation, we investigated the expression levels of several genes related to lipid metabolism including lipogenesis and *β*-oxidation. SREBP-1c has been shown to regulate the transcription of genes of lipogenic pathway [6]. Enzymes of the lipogenic pathway that are transcriptionally regulated include ACC1, FAS, and SCD1. ACC1 mediates the initial step of the fatty acid synthesis, and liver-specific ACC1 knockout mice show decreased hepatic triglyceride accumulation, suggesting that ACC1 plays a crucial role in the regulation of lipogenesis [27]. FAS catalyzes the last step in fatty acid biosynthesis, and thus, it is believed to be a major determinant of the maximal hepatic capacity to generate fatty acids by *de novo* lipogenesis [28]. SCD1 catalyzes the rate-limiting step in the production of monounsaturated fatty acids that are major components of tissue lipids [29]. Hence, it is suggested to the suppression SREBP-1c expression in liver may reduce the lipids accumulation. As a result of the low mRNA levels of SREBP-1c in livers of HFD-fed hamsters receiving zerumbone, there was a concomitant significant reduction in the mRNA expression of ACC1, FAS, and SCD1. Zerumbone is likely to have direct inhibitory effect on the SREBP-1c expression, which in turn influences the targeted lipogenic genes transcriptions, thereby reduces enzymes activity, resulting in a low rate of lipid synthesis. These results suggest that zerumbone can ameliorate HFD-induced hepatic steatosis via downregulation of lipid synthesis.

The accumulation of hepatic lipids by HFD could also be the result of a decrease in fatty acid *β*-oxidation [30]. Fatty acid *β*-oxidation takes place in two cellular organelles: mitochondria and peroxisome [31]. CPT-1 was chosen as markers of mitochondria, because they are rate-limiting enzymes of fatty acid *β*-oxidation and used to reflect mitochondria oxidation activity [32]. ACO was chosen as marker because it is a point to control peroxisomal *β*-oxidation [33]. The results showed that zerumbone increased the hepatic CPT-1 and ACO mRNA in HFD-fed hamsters, suggesting an enhancement of fatty acid *β*-oxidation. Therefore, we proposed that zerumbone could promote the catabolism and utilization of fat through inducing an increased expression of several genes involved in fatty acid *β*-oxidation.

As we know, the *β*-oxidation of lipids in liver could be regulated by different transcription factors. PPAR*α* is thought to be the principal regulator in the fatty acid oxidation [34]. The reduction of PPAR*α* expression in liver is indicative of impaired *β*-oxidation of fatty acids which may further influence the imbalance of lipid metabolism toward lipid accumulation in the case of induced lipogenic transcription. ACOX1 is the first and a rate-limiting enzyme of the PPAR*α* regulated and peroxisome proliferator-inducible fatty acid *β*-oxidation system [35]. To explore whether the effect of zerumbone on the attenuation of HFD-induced hepatic steatosis was related to PPAR*α* activation, mRNA expression of PPAR*α* and its target genes responsible for fatty acid *β*-oxidation were measured. Zerumbone markedly increased the HFD-induced low expression of hepatic PPAR*α* mRNA. Similar to the tendency in the target genes expression involving in CPT and ACO, ACOX1 mRNA expression in liver of HFD-fed hamsters were upregulated by zerumbone treatment. The same trend among these genes suggested that zerumbone enhanced *β*-oxidation in liver via the pathway of PPAR*α*-mediated gene transcription. Taken together, these results suggest that the effect of zerumbone on the treatment of hepatic steatosis may be partly associated with the enhancement of gene expression involved in lipid metabolism through PPAR*α* activation. 

**Figure 4:**
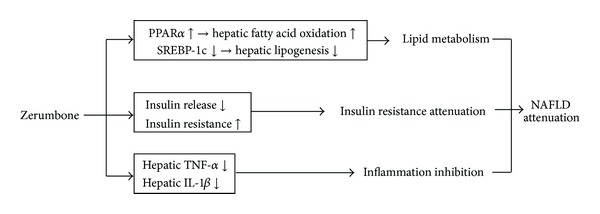
Possible mechanisms of zerumbone-mediated therapeutic activity in preventing and treating NAFLD.

In conclusion, our results show that zerumbone has a beneficial effect in inhibiting fat accumulation in liver, improves insulin resistance, inhibits inflammation, and possesses a repressive property on hepatic lipogenesis, which is associated with the inhibition of SREBP-1c and induction of PPAR*α*, suggesting a potential application of zerumbone in treating fatty liver diseases. Possible mechanisms of zerumbone mediated therapeutic activity in preventing and treating NAFLD has been shown in Figure 4. 

## Figures and Tables

**Table 1:** Summary of metabolic parameters in RCD- and HFD-fed hamsters receiving 8-week treatment.

	RCD fed	HFD fed				
	Vehicle	Vehicle	Zerumbone (mg kg^−1^ per day)			Lipanthyl
			75	150	300	(100 mg kg^−1^ per day)
Body weight (BW) (g)	124.37 ± 9.28^d^	167.90 ± 7.31^b^	161.84 ± 8.24^b^	157.33 ± 6.43^b^	150.61 ± 7.51^a,c^	141.72 ± 8.23^d^
Food intake (g day^−1^)	11.91 ± 3.26	12.08 ± 4.31	11.82 ± 4.06	11.97 ± 3.94	12.23 ± 3.23	12.16 ± 3.17
Water intake (mL day^−1^)	12.41 ± 2.13	13.12 ± 2.69	12.51 ± 2.24	12.78 ± 2.37	12.64 ± 2.83	12.23 ± 2.46
Liver weight (g 100 g^−1^ BW)	4.63 ± 0.22^c^	5.38 ± 0.31^a^	5.18 ± 0.29^a^	4.93 ± 0.34	4.85 ± 0.26	4.72 ± 0.25^c^
Plasma glucose (mmol L^−1^)	5.56 ± 0.26^d^	8.87 ± 0.24^b^	8.15 ± 0.19^b^	7.92 ± 0.21^b,c^	7.65 ± 0.23^b,c^	6.75 ± 0.18^a,c^
Plasma insulin (mU)	24.01 ± 0.19^d^	46.25 ± 0.38^b^	41.62 ± 0.42^b,c^	35.66 ± 0.36^b,c^	32.09 ± 0.27^b,c^	29.73 ± 0.35^a,d^
HOMA-IR	5.93 ± 0.16	18.23 ± 0.31^b^	15.07 ± 0.29^b,c^	12.55 ± 0.27^b,c^	10.91 ± 0.24^b,d^	8.92 ± 0.33^a,d^
Plasma TC (mmol L^−1^)	3.98 ± 0.18^d^	5.37 ± 0.23^b^	5.13 ± 0.28^b^	4.67 ± 0.22^b,c^	4.45 ± 0.18^a,d^	4.26 ± 0.21^d^
Plasma TG (mmol L^−1^)	0.43 ± 0.03^d^	1.33 ± 0.08^b^	1.18 ± 0.09^b^	1.01 ± 0.07^b,c^	0.86 ± 0.06^b,d^	0.67 ± 0.04^a,d^
Plasma LDL (mmol L^−1^)	2.71 ± 0.17^d^	4.25 ± 0.21^b^	3.99 ± 0.26^b^	3.54 ± 0.19^b,c^	3.29 ± 0.25^a,d^	3.05 ± 0.13^a,d^
Plasma HDL (mmol L^−1^)	1.17 ± 0.11^c^	0.85 ± 0.12^a^	0.90 ± 0.13^a^	0.93 ± 0.16^a^	0.98 ± 0.14	1.05 ± 0.16
Plasma FFAs (mmol L^−1^)	0.63 ± 3.1^d^	1.30 ± 4.7^b^	1.08 ± 3.9^b^	0.97 ± 3.5^b,c^	0.84 ± 4.1^a,d^	0.76 ± 3.8^d^
Hepatic TC (*µ*mol g^−1^ liver)	9.57 ± 0.38^d^	18.68 ± 0.61^b^	16.75 ± 0.64^b,c^	15.07 ± 0.57^b,c^	14.23 ± 0.45^a,c^	12.10 ± 0.53^a,d^
Hepatic TG (*µ*mol g^−1^ liver)	8.06 ± 0.39^d^	15.98 ± 0.61^b^	14.84 ± 0.57^b^	12.55 ± 0.41^b,c^	10.36 ± 0.55^a,c^	9.32 ± 0.49^a,c^

Zerumbone or lipanthyl was dissolved in distilled water for oral administration at the desired doses in a volume of 1.5 mL kg^−1^ once a day into HFD-fed hamsters. The vehicle (distilled water) used to dissolve the tested medications was given at the same volume. Values (mean ± SD) were obtained from each group of 8 animals after 8 weeks of the experimental period. ^a^ *P* < .05 and ^b^ *P* < .01 compared to the values of vehicle-treated RCD-fed hamsters in each group, respectively. ^c^ *P* < .05 and ^d^ *P* < .01 compared to the values of vehicle-treated HFD-fed hamsters in each group, respectively.
